# Joining Forces in Basic Science: ITS Meeting 2.0

**DOI:** 10.3389/ti.2022.10843

**Published:** 2022-09-26

**Authors:** Nina Pilat, Fadi Issa, Xunrong Luo, Anita Chong, Jonathan Bromberg, Katja Kotsch

**Affiliations:** ^1^ Department of Cardiac Surgery, Center for Biomedical Research, Medical University of Vienna, Vienna, Austria; ^2^ Transplantation Research and Immunology Group, Nuffield Department of Surgical Sciences, University of Oxford, Oxford, United Kingdom; ^3^ Department of Medicine, Division of Nephrology, Duke University, Durham, NC, United States; ^4^ Section of Transplantation, Department of Surgery, The University of Chicago, Chicago, IL, United States; ^5^ Department of Surgery, University of Maryland School of Medicine, Baltimore, MD, United States; ^6^ Department for General and Visceral Surgery, Charité Universitätsmedizin Berlin, Berlin, Germany

**Keywords:** transplantation, basic science, translational science, B cells, innate immunity, tolerance, biomarkers, big data

## Abstract

The second International Transplant Science (ITS) meeting jointly organized by the European Society for Organ Transplantation (ESOT), the American Society of Transplantation (AST), and The Transplantation Society (TTS) took place in May 2022 in one of Europe’s most iconic cities: Berlin, Germany. The ITS meeting 2022 was designed to serve as an international platform for scientific discussions on the latest ground-breaking discoveries in the field, while providing an excellent opportunity to present cutting-edge research to the scientific community. We think this is fundamental for the exchange of new ideas and establishment of collaborative work between advanced transplant experts, young professionals and early-stage researchers and students. Scientific sessions tackled hot topics in transplantation such as mechanisms of tolerance, biomarkers, big data and artificial intelligence. Our educational pre-meeting focused on the breakthrough and challenges in single-cell multimodal omics. The program included panel discussions illuminating various topics concerning conflicts and problems related to gender, such as challenges for female scientists. Attendees returned to their institutes with not only profound knowledge of the latest discoveries, technologies, and concepts in basic and translational science, but also inspired and excited after discussions and networking sessions with fellow scientists which have been duly missed during the pandemic.

Basic transplantation science meetings have always been small expert meetings designed to foster networks and collaboration between established scientists in the field and experts from other fields. The goal has always been to provide in-depth, cutting-edge talks from leading experts in the field, focusing on addressing challenges that arise from both, fundamental discoveries in basic science as well as clinical problems and unexpected hurdles in translational science. Even though there has always been a strong connection between basic science researchers in the field, the three international multidisciplinary societies (TTS, AST and ESOT) decided to join forces in organizing annual International Transplantation Science (ITS) meetings. The first ITS meeting was held in November 2019 in Clearwater, Florida [1]. When we started planning the subsequent meeting at the end of 2019, nobody could envision a global pandemic postponing almost all in-person meetings for 2 years. While planning this meeting we remained flexible and ready to respond to new challenges related to the pandemic, ultimately succeeding in creating a high-quality scientific program with cutting-edge topics, which the community acknowledged with a new record of 100+ abstract submissions.

The ITS meeting 2022 started with a pre-meeting workshop on “Single Cell Multimodal Omics,” an exciting technology which has recently gained a lot of attention in transplantation. Single-cell multimodal omics technologies can provide a “holistic approach” to study cells and tissues at the genomic, transcriptional and epigenomic level. Technical advances allow for the simultaneous assessment of multiple modalities as well as spatial organization, providing new opportunities in the discovery of new cell types, cellular differentiation trajectories and communication networks across cells and tissues. The session was started by Ricardo Ferreira (University of Oxford) who presented a multi-omics approach for simultaneous and targeted protein quantification increasing the power of single-cell RNA sequencing (scRNA-seq) to investigate heterogeneity of human T cell populations [2]. In the following talk, Matthias Farlik (Medical University Vienna) explained how scRNA-seq profiling can be used to assess disease progression and help with the discovery of novel biomarkers for monitoring before clinical symptoms arise. By monitoring genetic stability on a single cell and single strand level, Ashley Sanders (Max-Delbrück-Center Berlin) taught us how genetic mutations form and change cell states in health and disease. The final talk of this interesting session was given by Xunrong Luo (Duke University), discussing how these techniques are used for the discovery of novel cellular pathways in the rejection of kidney allografts.

For the main scientific sessions of the ITS meeting 2022 we created an innovative format aiming to put young researchers into the spotlight. Instead of having only invited talks for the thematic sessions, we selected additional and matching talks from abstract presenters, which were of outstanding scientific quality. The mix of educational talks from experts in their field and ongoing projects from younger researchers resulted in stimulating and inspiring discussions at the end of every session.

Session 1 focused on B cells and the role of protective/pathogenic antibodies in humoral alloresponses. Dr. Anita Chong (University of Chicago) set the stage for this interesting session by presenting her work on innate-like autoreactive B cells infiltrating kidney allografts. Using single-cell RNA sequencing it was shown that graft-infiltrating B cells exhibit an innate cell transcriptional state resembling mouse peritoneal B1 cells, which drive tissue destruction mediated by antibody-mediated rejection [3]. In the following talk, we switched to another organ and Emmanuel Zorn (Columbia University) provided insights into intragraft antibody responses in human heart allograft rejection [4]. His data revealed different expression profiles in transcriptomes of endomyocardial biopsies, indicating different types of antibody-mediated rejection. Oriol Bestard (University Hospital Vall d’Hebron) gave an update on his work of alloreactive memory B cells in kidney transplantation and its impact and implementation in the clinics. Three abstracts on B cells and antibodies were selected and discussed 1) the role of IgE after cardiac transplantation, 2) a novel immunosuppressive Bcl6-targeting compound for prevention of humoral rejection, as well as 3) (intragraft) donor-HLA-specific B cells in renal transplant patients.

The next session was all about “Big data and Artificial Intelligence (AI),” which are important topics, especially with implementation of –omics and sequencing techniques, both generating huge amounts of data. Sophie Limou (Nantes University) started with a talk on genomics in kidney transplantation, introducing the term “fat data” and raised awareness for biases and systematic errors in—omics studies. The next presentation from Kathie Connor (University of Edinburgh) was about machine learning in clinical transplantation. Although machine learning is increasingly important for transplantation research due to an increase of (very) big data sets and the increase in computer processing power and development of algorithms, there are lots of challenges and limitations about machine learning. Finally, we learned from Ali Zarrinpar about big data and AI in liver transplantation and how non-invasive techniques may supplement or even 1 day replace biopsies, which are the current standard of care for rejection assessment. Taken together, these talks provided lots of information on how to generate and interpret big data, and how AI can assist on the implementation of biomarkers in the clinic.

The final session of the day was on Innate Immunity and was kicked off with a presentation from Jonathan Bromberg (University of Maryland) on lymph node fibroblastic reticular cells and how these cells are able to steer immune responses. Afterwards, Andreas Diefenbach (Charité-Universitätsmedizin Berlin) spoke on Innate Lymphoid Cells (ILC) and how these populations are influenced by the gut microbiome.

The second day started with Session 5: Marginal Organs and *ex-vivo* machine perfusion. In this context, Zoltan Czygany (Universitätsmedizin Charité) reviewed recent findings on clinical machine liver perfusion. This presentation was followed by Cyril Moers (University of Groningen), who gave an excellent speech about machine perfusion in kidney transplantation, including updates of state-of-the-art and latest developments in the field to futuristic scenarios using cryopreservation strategies that would allow organ storage for longer periods of time and the development of “organ banks” for on-demand use. Another exciting strategy to decrease alloimmunity is silencing of HLA expression on donor cells, creating so-called “invisible organs.” Constanca Figueiredo (Medizinische Hochschule Hannover) reported on the success of her group with this approach using *ex vivo* machine perfusion to silence MHC transcription.

The following session focused on basic (T cell) immunology with the topic “Mechanisms of alloimmunity and tolerance.” The first speaker Ludger Klein (Ludwig-Maximilians University Munich), an expert in T cell development in the thymic micro-environment presented the “holy grail” in tolerance research and how MHC/peptide ligands are important for T cell repertoire selection. Gilles Blancho (Nantes University) gave updates on selective costimulation blockade and progress in the development of antagonist anti-CD28 therapeutics [5]. Two excellent abstracts presentations about how human regulatory macrophages can induce Treg generation and how Tregs are recruited to human kidney transplants after ischemia reperfusion injury were followed by a lively discussion among panelists and with the audience.

Session 7 was about COVID-19 and the immune response towards vaccination in transplant patients. Arne Sattler (Charite Universitätsmedizin Berlin) reported on findings about SARS-CoV2 vaccination of adolescent and adult kidney transplant recipients and their outcomes based on comprehensive humoral-, B- and T cell analyses. His data document that kidney transplant patients constitute a special patient group, which needs to be carefully evaluated after vaccination as the immune response is dampened mainly due to antimetabolite treatment [6, 7]. Afterwards Petra Bacher (Christian-Abbrechts University Kiel) discussed low-avidity CD4^+^ T cell responses to SARS-CoV-2 in unexposed individuals and humans with severe COVID-19. She demonstrated that SARS-CoV-2-reactive CD4^+^ memory T cells were present in unexposed individuals, displaying low functional avidity and multiple, highly variable cross-reactivities, e.g., towards common cold coronavirus, which were not present COVID-19 patients.

The keynote lecture was given by Florent Ginhoux (Singapore Immunology Network). He reported on single cell profiling strategies to characterize myeloid cells in health and disease [8].

The last day of the meeting started with “Basic mechanisms of organ regeneration and organoids” and an excellent talk given by Luc van der Laan (Erasmus University Medical Centre) on organoids and liver regenerative medicine.

The next session “A star is born” was designed to bring the very best young researchers on stage, so the best ranked abstracts had the chance of being presented in front of a big audience and the presenters showing off not only their data but also their presentation skills in front of a tough jury.

The last session was about Biobanks and Bioassays and our first speaker was Sarah Cross (University of Oxford), who is not only manager of the Oxford biobank but also National Coordinator for Quality in Organ Donation (QUOD), an initiative that aims to improve and facilitate the collection of biological samples in order to improve the understanding of all aspects of organ donation and transplantation. Jianing Fu (Columbia University) gave insights into her and Megan Sykes’ work on the alloreactive T cell repertoire in transplant patients and how to track these cells by TCR sequencing. Federica Genovese (Nordic Bioscience Copenhagen) presented a novel bioassay to determine the activity of extracellular matrix remodeling and how this can be used for a more accurate prognosis in kidney transplant recipients in the future.

Overall, we had a vibrant and inspiring meeting after a long period of “scientific isolation.” Hungry for more, we look therefore forward towards the next meeting which will be organized by TTS in 2023, to be held in North America.
